# Editorial: Stem Cells and Aging

**DOI:** 10.3389/fnagi.2021.690613

**Published:** 2021-05-17

**Authors:** Cesar V. Borlongan, Gary K. Steinberg

**Affiliations:** ^1^Department of Neurosurgery and Brain Repair, University of South Florida, Tampa, FL, United States; ^2^Department of Neurosurgery, Stanford University, Stanford, CA, United States

**Keywords:** stem cells, aging, transplantation, brain, regeneration

Stem cells have emerged as a key scientific tool and potent therapeutic agent for both understanding the mechanism of aging, as well as treating diseases that arise as cells lose their resilience in later years of life. Science continues to advance our comprehension and capacity to harness the innumerable possibilities that stem cells hold for ameliorating disease burden and improving the quality of life. Collaborative efforts across the globe have helped elucidate the role of stem cells in reversing the consequences of age-related neurologic disorders, including stroke, Parkinson's disease (PD), and Alzheimer's disease (AD). This special issue is a testimony to the enthralling breakthroughs over the past year in stem cell therapy for aging-related disorders.

PD manifests as neurodegenerative destruction of dopaminergic neurons in the substantia nigra pars compacta due to pathological accumulation of misfolded alpha-synuclein inclusions. This aberrant protein aggregation precipitates disabling motor deficits and poor quality of life in millions of patients globally. Dr. Zhiping Hu of Central South University, an innovative mind in the field of ischemic stroke research, has also led critical efforts to advance our understanding of PD in the laboratory and the literature. In the present issue, Liu et al. clarify the importance of bone marrow stromal cells in PD models by investigating the pooled effects observed in 27 major animal studies to date.

Spinal cord stimulation (SCS) is a promising focus of PD research that leverages implantable electrodes to deliver controlled electrical signals to the spine. This represents an alternative therapeutic approach to traditional pharmacological options that mirrors concepts inherent to deep brain stimulation, another treatment that demonstrates success in certain PD patients. Dr. Isao Date of Nagoya City University is a world-renowned researcher who has contributed crucial data to the PD field. Here, Kuwahara et al. present novel findings that continuous SCS effects positive behavioral and histological changes in PD. The principles outlined in this study contain tremendous translational value that underscores the potential utility of SCS in the clinical setting.

Human umbilical cord blood cells (HUCBCs) signify a potent source of stem cells that have demonstrated advantageous outcomes across a diverse array of conditions. Dr. Jieli Chen is an esteemed stroke expert from the Henry Ford Hospital who has leveraged HUCBC stem cells in her research to contribute compelling evidence for the neuroprotective efficacy of these cells against devastating conditions such as stroke. In the accompanying article, Venkat et al. set the stage for the use of HUCBCs in vascular dementia. They provide compelling evidence of white matter remodeling with cognitive functional improvements that establish HUCBCs as a potentially robust therapeutic tool.

Dr. Stefano Pluchino of the University of Cambridge has led pioneering studies on developing experimental molecular stem cell-based medicines designed to transform our understanding of the mechanisms of intercellular neuro-immune signaling into therapeutics for multiple sclerosis (MS) and other inflammation-plagued disorders, such as stroke and traumatic brain injury. Here, Nicaise et al. discuss the regenerative capabilities of the resident stem cells in the aging brain. They investigate factors that influence neural stem cell aging and provide both *in vivo* and *in vitro* models to illustrate their findings.

Limited data have supported a critical time period for menopausal women with AD to undergo estrogen replacement therapy (ERT). Dr. Sha Sha is a senior lecturer at Nanjing Medical University with many years of experience in the field. Here, Qin et al. elucidate the effective window of estradiol treatment utilizing an AD mouse model. They examine β-amyloid accumulation, telomerase activity, hippocampal neurogenesis, hippocampal dependent behavior and neural stem cell (NSC) proliferation to discover an effective window for treatment.

The utilization of stem cells has provided a potent therapeutic for the treatment of central nervous system disorders. He et al. boasts years of experience as a cerebrovascular neurosurgeon and scientist focused on translating key scientific findings to the clinic. Here, Dr. Steinberg of Stanford University and colleagues discuss past and current clinical trials utilizing stem cell-based therapy as a treatment for ischemic stroke. Specifically, they discuss the recurring use of hematopoietic, mesenchymal, and neural cell lineages and the reasoning behind this.

Stroke is a debilitating illness with limited therapeutic options. Dr. Yumin Luo is a leader in the field of cerebrovascular disease from Capital Medical University, who specializes particularly in stroke. Here, Fan et al. explored the role of long non-coding RNAs (lncRNAs) in the pathology of ischemic stroke and intracerebral hemorrhage. The authors explicate the diagnostic and therapeutic capacity of lncRNAs in stroke and the potential for stem cells to further bolster lncRNA function.

Aging-induced deterioration of olfactory sensory neurons in the olfactory epithelium (OE) can be alleviated by promoting OE regeneration through certain chemical factors. Dr. Yiqun Yu has led remarkable studies investigating the molecular mechanisms of the olfactory bulb. Li et al. at Fudan University found that levels of G-protein coupled receptor 5 (Lgr5)-positive cells were reduced substantially in the injured OE of aged mice, and Notch signaling promoted OE regeneration.

The neuronal transcription factor NeuroD1 has been shown to promote the differentiation of neurons (iNeurons) from astrocytes following a stroke, suggesting a potential regenerative therapy. Dr. Ling Wei from Emory University has led innovative studies investigating neuroprotective agents in stroke. Here, Jiang et al. found that NeuroD1 administration *via* lentivirus culminated in the transformation of the infected astrocytes into iNeurons in mice, which rehabilitated impaired neuronal circuitry induced by stroke.

These milestone studies in basic science, translational medicine, and clinical research demonstrate the therapeutic potential of stem cells in aging-related neurological disorders (Figure 1). The present pandemic highlights age as a primary factor driving mortality and morbidity. Age serves as a significant determinant of health not just for COVID-19, but for most of the diseases that we endeavor to treat. Our scientific efforts to understand the mechanisms of cell death and survival in the brain toward therapeutically harnessing healthy aging will benefit from the rigorous investigations into the clinical applications of stem cells.

**Figure 1:**
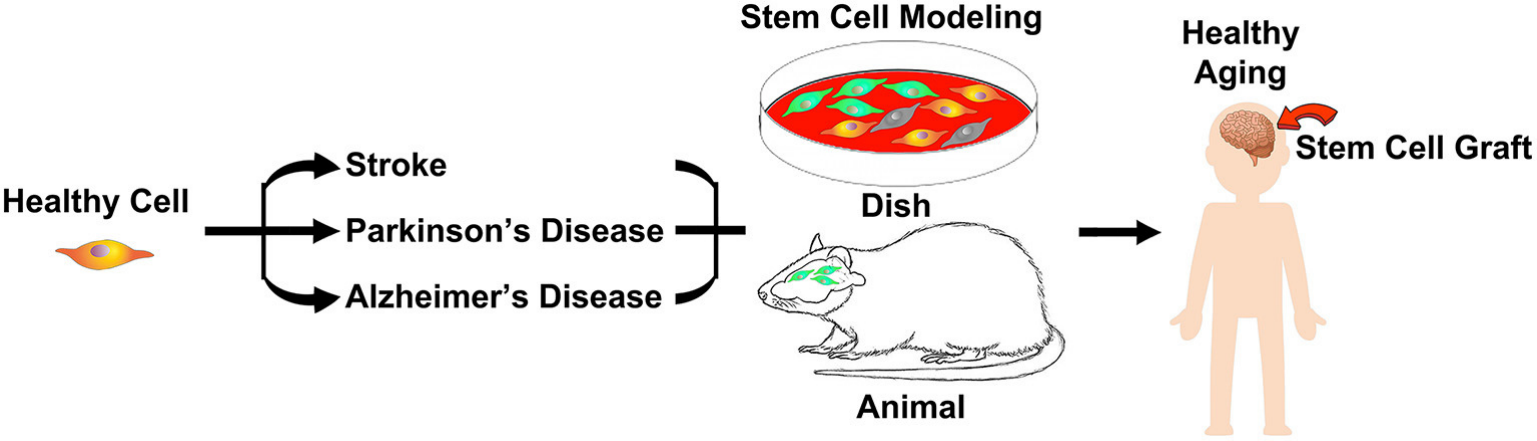
During aging and disease states, such as stroke, Parkinson's disease and Alzheimer's disease, brain cells become vulnerable to injury, which can be captured in a pertri dish or animal models. Stem cell therapy stands as a robust approach in harnessing healthy aging.

## Author Contributions

All authors conceptualized and wrote the manuscript.

## Conflict of Interest

CB and GS serve as consultants to stem cell-based companies, hold patents and patent applications, and have funded grants related to stem cell biologics and applications.

